# An individual-based model for collective cancer cell migration explains speed dynamics and phenotype variability in response to growth factors 

**DOI:** 10.1038/s41540-017-0006-3

**Published:** 2017-03-03

**Authors:** Damian Stichel, Alistair M. Middleton, Benedikt F. Müller, Sofia Depner, Ursula Klingmüller, Kai Breuhahn, Franziska Matthäus

**Affiliations:** 1 BIOMS/IWR, University of Heidelberg, Im Neuenheimer Feld 267, Heidelberg, 69120 Germany; 2 DKFZ Heidelberg, KKE Neuropathologie, Im Neuenheimer Feld 221, Heidelberg, 69120 Germany; 3 Institute of Pathology, University Hospital Heidelberg, Im Neuenheimer Feld 221, Heidelberg, Germany; 4 Translational Lung Research Center (TLRC), Member of the German Center for Lung Research (DZL), Heidelberg, Germany; 5 CCTB, University of Würzburg, Campus Hubland Nord 32, Würzburg, 97074 Germany; 6 FIAS, University of Frankfurt, Ruth-Moufang-Str. 1, Frankfurt am Main, 60438 Germany

## Abstract

Collective cell migration is a common phenotype in epithelial cancers, which is associated with tumor cell metastasis and poor patient survival. However, the interplay between physiologically relevant pro-migratory stimuli and the underlying mechanical cell–cell interactions are poorly understood. We investigated the migratory behavior of different collectively migrating non-small cell lung cancer cell lines in response to motogenic growth factors (e.g. epidermal growth factor) or clinically relevant small compound inhibitors. Depending on the treatment, we observed distinct behaviors in a classical lateral migration assay involving traveling fronts, finger-shapes or the development of cellular bridges. Particle image velocimetry analysis revealed characteristic speed dynamics (evolution of the average speed of all cells in a frame) in all experiments exhibiting initial acceleration and subsequent deceleration of the cell populations. To better understand the mechanical properties of individual cells leading to the observed speed dynamics and the phenotypic differences we developed a mathematical model based on a Langevin approach. This model describes intercellular forces, random motility, and stimulation of active migration by mechanical interaction between cells. Simulations show that the model is able to reproduce the characteristic spatio-temporal speed distributions as well as most migratory phenotypes of the studied cell lines. A specific strength of the proposed model is that it identifies a small set of mechanical features necessary to explain all phenotypic and dynamical features of the migratory response of non-small cell lung cancer cells to chemical stimulation/inhibition. Furthermore, all processes included in the model can be associated with potential molecular components, and are therefore amenable to experimental validation. Thus, the presented mathematical model may help to predict which mechanical aspects involved in non-small cell lung cancer cell migration are affected by the respective therapeutic treatment.

## Cancer research: Mathematical model describes mechanics of cell coordination

In many cancers, spreading and the formation of metastasis involve the coordinated migration of many cells. An interdisciplinary team of researchers from Heidelberg and Frankfurt studied the collective movement of cultured lung cancer cells subject to chemical stimulation. Based on extensive data analysis a mathematical model was developed to explain the variety of migration behaviors observed under different treatments. The model describes the mechanics of compression, stretch, cell elasticity and force-regulated active motion—which in sum lead to coordination within large cell groups. Simulations demonstrate how these mechanical features affect cell coordination and collective behavior. In tests of potential medical treatment strategies, the model can be used to predict the effects of the drug on specific mechanical properties of single cells.

## Introduction

Cell migration is essential for morphogenesis or tissue regeneration under physiological conditions.^1^ However, under patho-physiological conditions such as tumorigenesis, cell motility may cause dissemination of malignantly transformed cells, which correlates with poor survival and early recurrence in many solid epithelial carcinomas. The relevance of tumor migration and cell dissemination for patient outcome is indicated by the clinically important TNM staging system. This system describes the stage of a tumor based on its size (T0–T4) and the presence of regional lymph node metastasis (N0–N3) and distant metastasis (M0/1). Especially the N and M staging are directly linked to the migratory ability of tumor cells. This staging is not only important for treatment decisions but also for the prognosis of patients.^2, 3^ Other studies confirm a positive correlation between the number of lymph node metastasis, which can only originate from mobile tumor cells, and a reduction in the 5-year survival rate of patients. Further, the prognosis of patients after surgery directly correlates with the number of lymph node metastases,^4^ and thus the number of tumor-positive lymph nodes represents an independent prognostic marker for non-small cell lung cancer (NSCLC) patients.^5^ Very recent findings indicate that next to ‘conventional’ tumor spread through the blood stream, a new mechanism supporting tumor spread might occur. Spread through air spaces (STAS) promotes tumor cell dissemination in about 50% of lung adenocarcinoma patients. Importantly, STAS is significantly associated with and its occurance correlates with poor overall survival of adenocarcinoma patients.^6^ However, controlling metastasis in a therapeutic setting by targeting individual cellular processes (i.e. cell adhesion and perturbation of relevant signaling pathways) is challenging as cancer cells exhibit a large diversity of mechanisms supporting tumor cell dissemination.^1^

At the cellular level, epithelial cancer cells exhibit different modes of motility including sheet migration, migration of smaller cell clusters, or of individual mesenchymal-like cells,^7^ and first molecular mechanisms discriminating between these phenotypes have been identified.^8, 9^ These different types of motility are induced and modulated by extracellular stimuli including secreted growth factors (e.g. hepatocyte growth factor (HGF), epidermal growth factor (EGF)) or extracellular matrix (ECM) components. Indeed, dysregulation of signaling pathways and subsequent induction of tumor cell dissemination is frequently observed in human carcinogenesis.^10^ Interestingly, pathological examinations and experimental data illustrated that many epithelial cancers favor collective cell migration and that tumor cell clusters exhibited increased metastatic potential.^10^ However, it is not fully understood how mechanical properties such as intercellular forces between tumor cells affect this clinically relevant migratory phenotype.

Epithelial cancer cells often require intact cell–cell junctions for survival and efficient migration. In case of collective migration, usually investigated by lateral migration into a defined gap, the mechanical stimulation, loss of contact inhibition of marginal cells, and stimulation by growth factors can induce directed migration towards cell-free areas. Hereby, marginal cells, forming the tissue front, are believed to stimulate polarization in subsequent cell layers,^11, 12^ the submarginal cells which are adjacent to the marginal cells, and bulk cells far away from the tissue front. This is supported by studies showing that cells polarize in the direction of applied stress.^13, 14^ In addition, sub-marginal cells have been shown to extend ‘cryptic’ protrusions underneath their anterior neighbors^15^ and to actively contribute to directed migration.^16, 17^ Studies involving normal and malignantly transformed cell types illustrate that these effects do not only depend on growth factor stimulation but also on adhesive forces and cell–cell contact.^10, 18, 19^

Several biological processes underlying collective cell migration have been formulated in terms of mathematical models that describe cell–cell interactions and the emergence of collective phenomena. Vicsek et al. proposed a simple model describing the displacement of cells according to inter-cellular forces and active migration exhibiting directional persistence.^20–22^ Recent variations of this model involved features such as velocity alignment between neighboring cells,^23, 24^ force-velocity alignment,^25^ and lateral drag.^11^ Apart from these center-based models, other approaches explicitly include the cell shape. These comprise Cellular Potts Models,^23, 26, 27^ where each cell is represented by a set of connected points on a lattice, vertex-based models,^28^ where the boundary of the cell is given as a set of vectors, or models based on phase fields with more details on the single-cell level, such as deformable boundaries, actin-dependence or cell contraction.^29^ In general, cell-based models allow for a large amount of detail, such as the inclusion of signaling network dynamics, the coupling with external chemical or mechanical gradients, cell proliferation and apoptosis.^30^ Because of this, cell-based models are in general very powerful tools to describe many different biological processes.

In this study we focused on the variations in the motility of cells and the cell–cell interactions under different treatment conditions. The experimental setup involved cells from lung cancer cell lines, positioned on a 2D surface without ECM coating or external gradients, which were observed for two to three days. In addition, proliferation, which might affect the biological read-out of migrating cells, was blocked in a subset of the experiments using mitomycin-C. To model this setup, we chose a center-based off-lattice cell model, in which only the center position of each cell is considered, and subject to inherent or imposed movement. Even though the cell shape was not explicitly considered in the model, it can be derived through a Voronoi tessellation based on the neighborhood structure given by the Delaunay triangulation. The model we are presenting here consists of only two equations for each cell, and 8 parameters. It is thus an extremely simple model of low computational cost. It allows the modeling of a large number of interacting cells, and also facilitates mathematical tractability (see Discussion).

The existing center-based models on collective cell migration were all designed to reproduce features connected to the evolution and coordination of the *orientation* of cellular velocity. However, a detailed mathematical description of speed dynamics (e.g. acceleration) induced by physiologically relevant growth factors or therapeutic perturbation approaches (e.g. receptor kinase inhibitors) as well as the interplay between stimulation and mechanical cell–cell interaction was missing.

In our study, we present an individual-based model that is capable of reproducing stimulus-induced speed dynamics, as well as diverse front phenotypes observed in experimental data sets derived from lung cancer cell lines (non-small cell lung cancer; NSCLC) after stimulation and perturbation of pro-migratory signaling pathways. The model demonstrates that the existence of a non-specific mechanotransduction mechanism is necessary and sufficient for all observed phenomena, but also shows that the effect of treatment on the individual cells cannot be directly inferred from the speed dynamics or from the front phenotype.

## Results

### Stimulation and cell density define the mode of lung cancer cell migration

To analyze the impact of pro-migratory stimuli (e.g. growth factors) on the migratory behavior of epithelial cancers and to generate a corresponding mathematical model we focused on cell lines derived from human non-small cell lung cancer (NSCLC, subtype adenocarcinoma) since tumor cell dissemination represents a clinically relevant prognostic marker for NSCLC patients.^31^ Different NSCLC cell lines (H1975, H1650) showing predominant collective migration were selected for the analysis of lateral migration into a defined 500 µm gap after stimulation with potent motogens (HGF), (EGF), insulin-like growth factor (IGF)-1, and fetal calf serum (FCS). The biological effects of these stimuli were monitored using time-lapse microscopy for up to 72 h. In total, more than 120 videos of NSCLC cells were analyzed for this study (Table 1).

**Table 1:** Phenotype classification for 123 time lapse movies of H1975 and H1650 cells after visual inspection

	

Shown are the numbers of time-lapse videos for each category, e.g. the number of replicates for each treatment showing a given phenotype (in 20 replicates with FCS stimulation, 19 show straight fronts, 1 shows cellular bridges). The gray shading indicates the relative distribution into the different phenotypes for every treatment (out of 20 FCS 95% of replicates show straight fronts). *Dark shading* indicates high percentage and vice versa

**Fig. 1:**
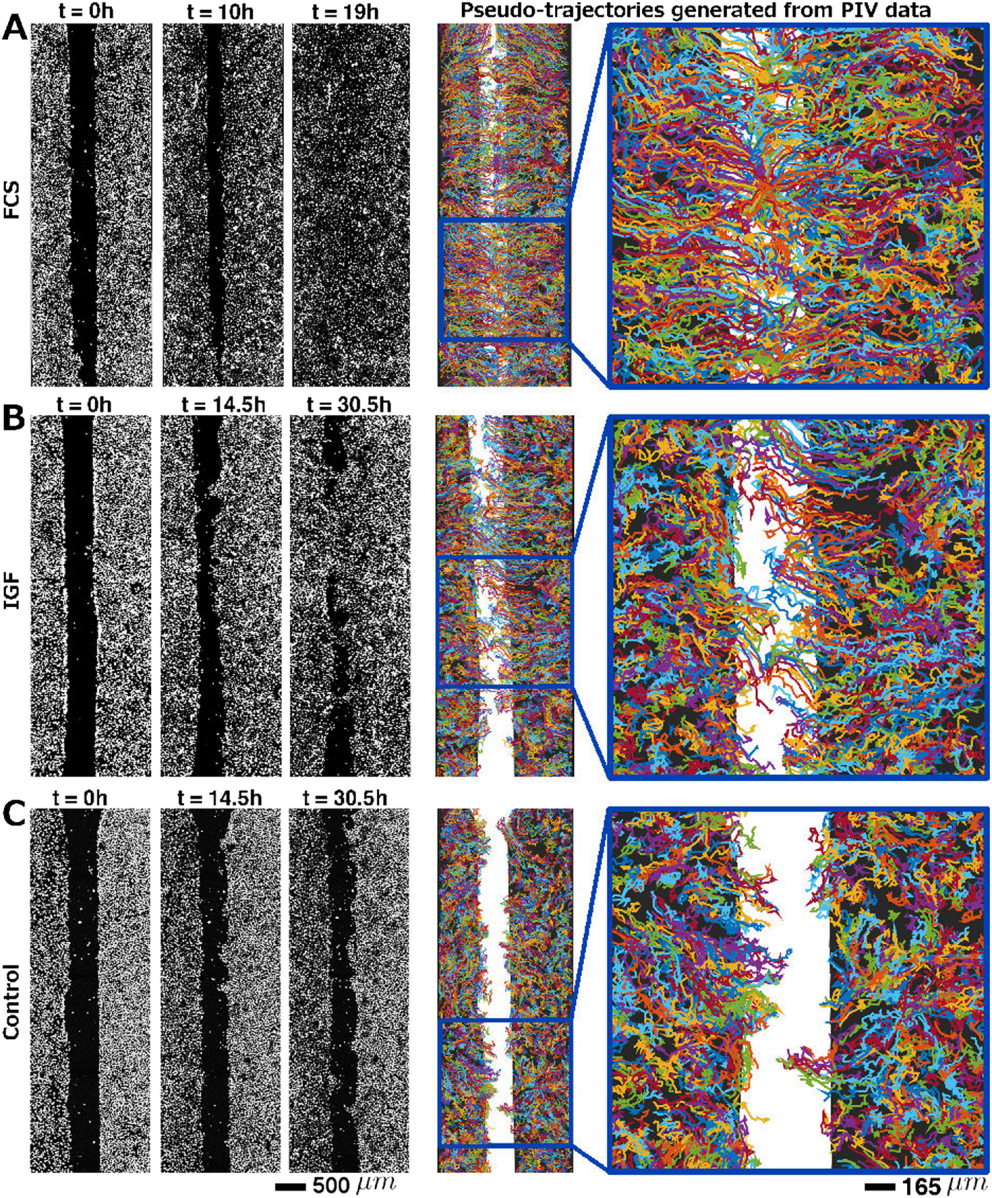
Different phenotypes of sheet migration in NSCLC H1975 cells associated with different treatments. (**a**) Straight front after treatment with FCS. (**b**) Cellular bridges after treatment with IGF. (**c**) Finger shapes/undulating fronts in a control experiment (no stimulation). Snapshots are showing stained nuclei at time points *t* = 0, 10, and 19 h (**a**), and at time points *t* = 0, 14.5, and 30.5 h (**b**, **c**). On the right, pseudo-trajectories generated from PIV data are shown for each experiment for the entire field of view and an enlarged section. Random coloring of the trajectories has been chosen for better visibility of the paths of neighboring cell groups. The pseudo-trajectories show clearly that after stimulation with FCS also bulk cells far from the front engage in directed migration towards the gap. After stimulation with (IGF)-1 cells further than 500 μm do not engage in directed migration. For unstimulated cells (**c**) persistent migration towards the gap is almost entirely absent. Original movies for a, b and c are provided as Supplementary Material

**Fig. 2:**
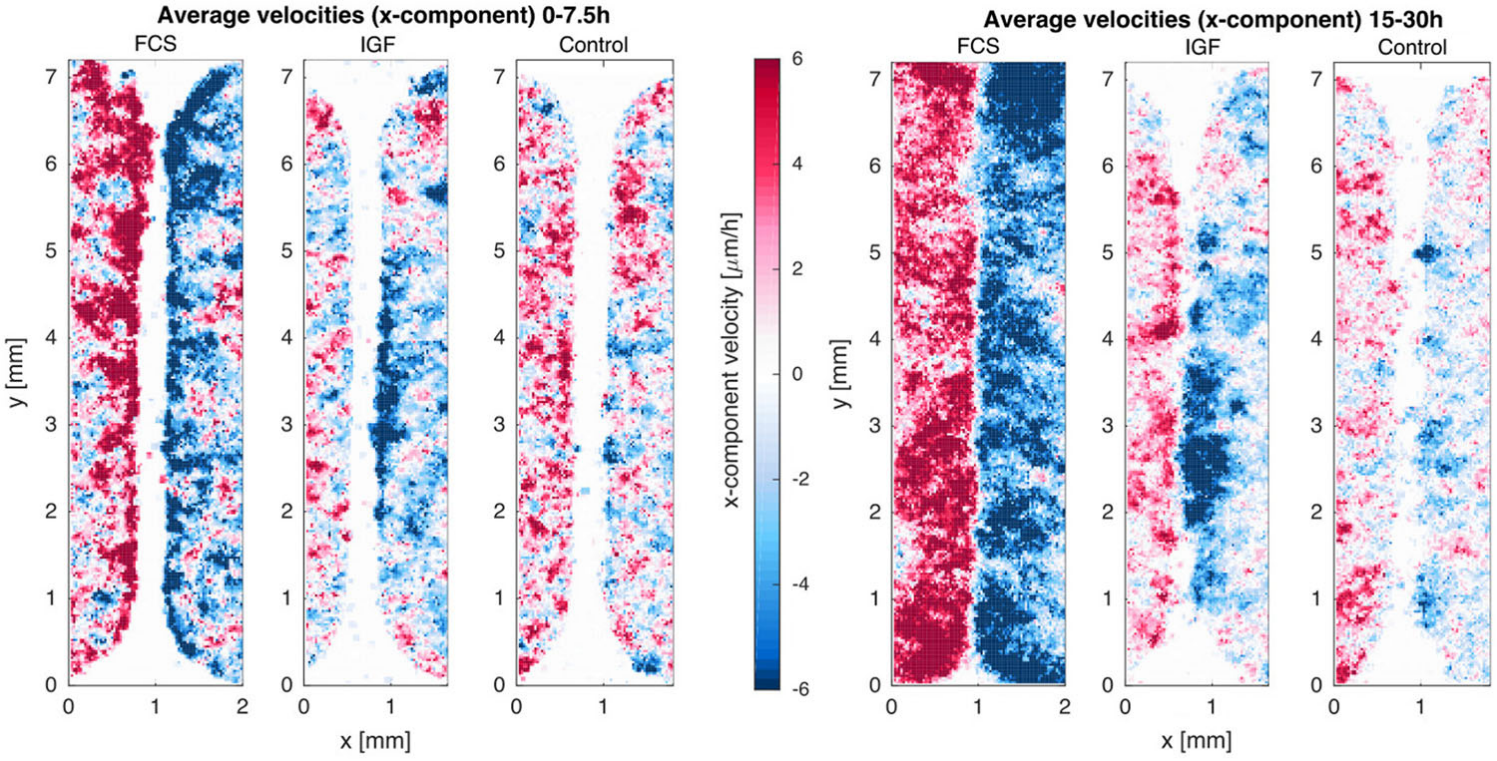
Migration activation pattern derived from PIV for the unstimulated, FCS or IGF stimulated H1975 cells displayed in Fig. 1. The activation maps show the time average of the spatially resolved x-components of the velocity field for an early time span (averaged over the first 7.5 h) (**a**), and a later time span (15–30 h average) (**b**). These maps illustrate that directed motility is first activated at the front and then back-propagates into the submarginal and bulk cells. The level of front activation and the degree to which activation spreads into the tissue is modulated by the treatment

Interestingly, we observed different gap closure phenotypes of the cell monolayers, which were associated with the different treatments (Table 1). While FCS resulted in a fast and efficient propagation of straight cell fronts (Fig. 1a), growth factors induced partial gap closure and formation of cellular bridges between the major cell populations, as shown for IGF stimulation in Fig. 1b. When the tumor cells were deprived of growth factors the front developed an irregular shape and inward migration was restricted to certain areas resulting in undulating fronts (Fig. 1c). Pseudo-trajectories (Fig. 1, right panels), derived from particle image velocimetry (PIV) (see Methods), further illustrate that treatment with growth factors stimulated coordinated persistent migration towards the gap area, and that the strength of stimulation was associated with the activation of a larger fraction of cells (Fig. 2). After FCS stimulation also bulk cells far away from the monolayer front engaged in persistent migration, while with IGF treatment exclusively cells in close proximity to the gap (around 500 μm) showed active migration (Fig. 2).

All three phenotypes (straight fronts, cellular bridges and undulating fronts) were exclusively associated to cell migration since inhibition of proliferation by mitomycin-C did not affect the observed cell behavior (see Supplementary Materials). Next to the different treatments, cell density appeared to be an additional factor determining the migratory behavior. For high cell densities, straight fronts and effective gap closure were frequently detected. In contrast, low cell densities were connected with reduced migration and the formation of cellular bridges or finger-shapes (see Supplementary Materials).

In summary, the administration of full cell culture media or with single pro-migratory growth factors and the cell density define the front phenotype of lung tumor cells in vitro.

### PIV analysis reveals characteristic speed dynamics after stimulation of NSCLC cell lines

In order to capture a large section of the gap and to avoid bleaching effects, images were taken at low magnification and frame rate. This resulted in image sequences, which made segmentation of individual cell nuclei and single cell tracking impossible. We therefore used PIV^32, 33^ to obtain quantitative information on the spatio-temporal velocity distributions and to characterize the effects of the different treatments on the migratory behavior of the cells of the NSCLC cell lines. PIV proved to be computationally efficient and robust. Because PIV does not rely on single cell segmentation, also phase contrast movies could be analyzed.

**Fig. 3:**
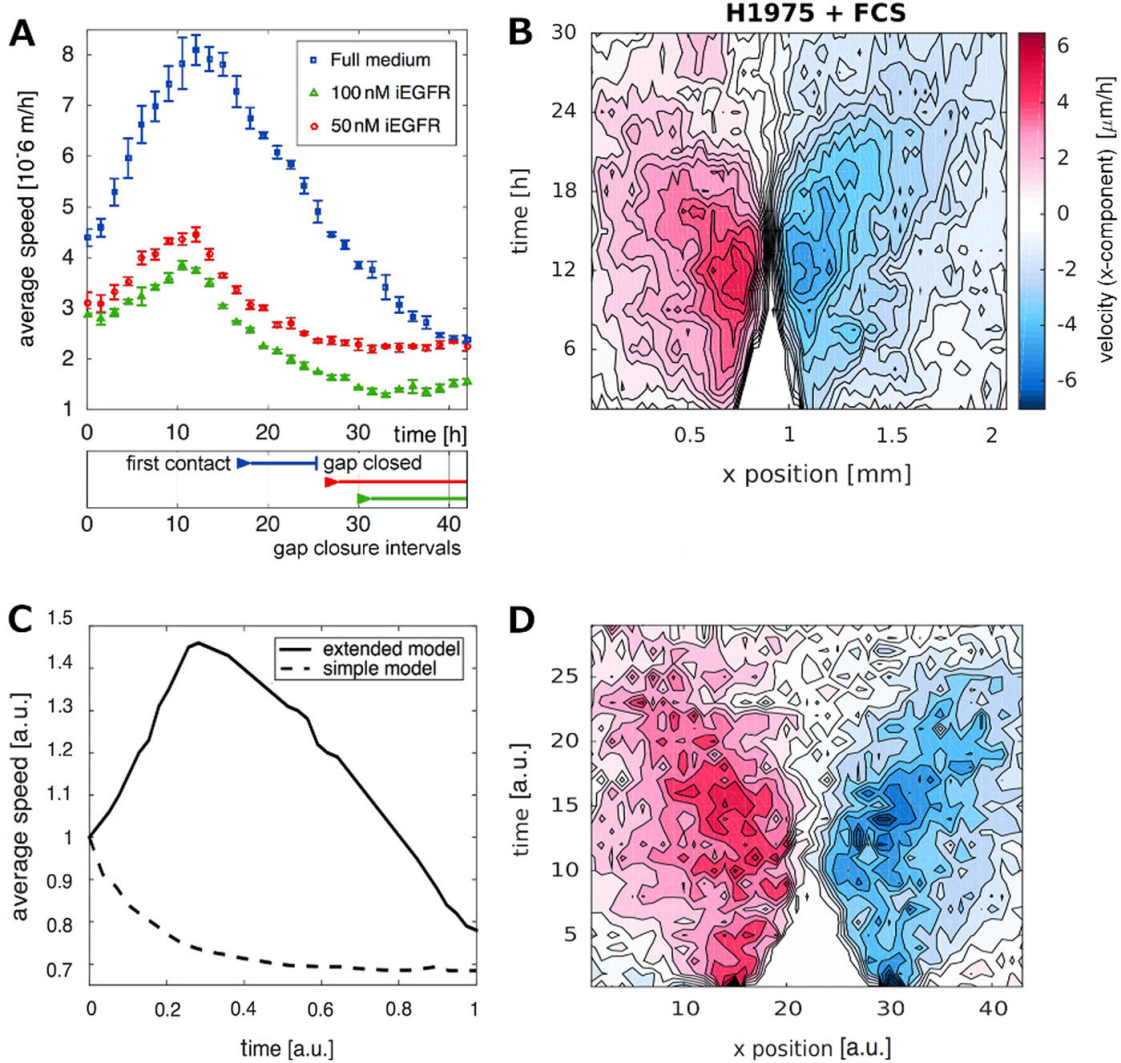
Speed dynamics and speed kymographs derived from PIV and simulations. (**a**) Characteristic speed dynamics of H1975 cells treated with different doses of iEGFR. Lateral migration of NSCLC cells (H1975) was quantitatively measured for 40 h using time-lapse microscopy after treatment with full medium (containing FCS) or kinase inhibitors at different concentrations (iEGFR; erlotinib with 50 and 100 nM). Error bars represent the standard error from three independent replicates. Gap closure intervals (from first contact of the opposite fronts to complete gap closure) are indicated below. Note that the characteristic speed dynamics, initial increase—peak and following decay, is seen for all treatments. The movies from which the data is derived are provided in the Supplementary Materials. (**b**) PIV-derived speed kymographs for an experiment involving H1975 cells stimulated with FCS. Shown are velocity components perpendicular to the gap displayed as a contour plot. *Colors* indicate displacements in *x*-direction with values ranging from −6 μm/h (negative *x*-direction, *dark red*) to 6 μm/h (positive *x*-direction, *dark blue*). *White* color indicates zero speed, i.e. in the gap area (the *white triangle*). (**c**) Typical average speed dynamics of cells in a simulation with and without directed migration. The simple model without directed motility (Eq. 3) yields only monotonously decaying speeds lacking the characteristic peak. The extended model including directed motility (Eq. 4) qualitatively reproduces the characteristic profile seen in all experiments with initial acceleration and subsequent deceleration, and also the relative increase in the speeds with respect to the initial value. Here speed values are scaled with respect to the initial value at *t* = 0, i.e. maximal average speeds in the simulation reach 150% of the initial value, which agrees well with the experimental data. (**d**) The velocity kymograph (only *x*-component, *red*: high velocities in positive *x*-direction, *blue*: high velocities in negative *x*-direction, *white* codes for speeds = 0) of simulated cells shows a similar butterfly-shaped spatio-temporal speed activation as typical in the experiments. Parameters for (**c**) and (**d**) were chosen to achieve straight fronts and complete gap closure. In particular, we used *D* = 0.001, *F*_0_ = 1, *a* = 1.818, *r*_*c*_ = 0.0333, *σ* = 1.2, *A*_0_ = 20, *τ* = 0.0988, *θ* = 1. Note that the images are only intended to show qualitative agreement between simulation and data, like the acceleration-deceleration profile of the speed evolution, agreement in the relation between maximal and initial speeds (simulations: 1.5 fold, experiments: 1.5–2 fold), the butterfly-shaped activation pattern, or the linear dynamics in the gap closure. The velocities in the simulations are not comparable with the data in a quantitative manner, since the model is given in scaled and dimensionless variables and parameters

In all experiments the absolute speeds, averaged over the entire field of view, increased over a time interval of several hours and decreased afterwards. Pro-migratory treatment was associated with a stronger and prolonged acceleration phase (Fig. 3a). The peak amplitude was also associated with the duration until gap closure. However, the duration of the acceleration phase was not determined by (and not correlated to) the timing of gap closure. The speed dynamics showed peaks also for experiments where the gap did not close at all (e.g. after treatment with the EGF receptor inhibitor erlotinib, Fig. 3a).

The spatio-temporal velocity distribution showed high velocity components directly at the front for early time points, which then extended towards submarginal and bulk cells (Fig. 3b). The largest contribution originated from the velocity component perpendicular to the gap. Often, the acceleration wave spread only to some extent into the monolayer, and bulk cells far away from the front did not migrate significantly. In summary, different treatments were associated with different gap closure phenotypes.

However, PIV analysis showed that the spatio-temporal speed dynamics were qualitatively similar for all experiments with characteristic initial acceleration and subsequent deceleration. Also the spatio-temporal speed distribution was similar for all experiments. These results lead to the following questions: (i) which features on the single cell level give rise to the experimentally observed front phenotypes of the cell monolayer? In particular, can the observed range of migratory behaviors and the spatio-temporal velocity distribution be explained by simple assumptions on the (mechanical) properties and interaction of the individual cells? And (ii) how do treatments affect the properties of individual cells such that different front phenotypes develop?

### Establishment of a mathematical model reflecting different phenotypes induced by different stimuli

In order to answer these questions, we developed an individual-based model that defines a small set of cellular features that reproduce and explain the observed velocity distributions, front phenotypes and many other features of the collective migration. Our approach is based on a recently developed simple model of cell migration, combining random motility and cell-cell adhesion.^34^ The model is given in dimensionless and scaled variables (see Supplementary Materials) and describes the displacement of each cell as a response to external forces exerted from neighboring cells:

1 $${{\boldsymbol{F}}}_{i,k}={F}_{0}\cdot F(\Vert {{\boldsymbol{x}}}_{i}-{{\boldsymbol{x}}}_{k}\Vert )\frac{{{\boldsymbol{x}}}_{i}-{{\boldsymbol{x}}}_{k}}{\Vert {{\boldsymbol{x}}}_{i}-{{\boldsymbol{x}}}_{k}\Vert },$$

with *F*_0_ a positive constant, indicating the strength of the mechanical interaction, ***x***_***i***_ = (*x*_*i*_, *y*_*i*_) the position of cell *i*, and

2 $$F(r)=\left\{\begin{array}{ll} 2\cdot ({e}^{-2a(r-{r}_{c})}-{e}^{-a(r-{r}_{c})}), & r < \sigma {r}_{c}\\ 0, & r\ge \sigma {r}_{c}\end{array}\right..$$

With this description the force between two cells is positive (repulsive) when their distance is below *r*_*c*_, and negative (attractive) for *r* > *r*_*c*_. Repulsion accounts for the fact that the cells are compressible only to a certain degree. Attraction reflects cell–cell adhesion and yields correlated motion of neighboring cells. The attractive part of the potential acts only in the neighborhood *σr*_*c*_ of the cell. If cells are further apart from each other than *σr*_*c*_, they do not exert force on each other and show uncorrelated migration. The radius *r* = *r*_*c*_ reflects the natural cell size where attraction and repulsion balance. The parameter *a* quantifies cell elasticity: cells are compressible for small *a* and stiff for large *a*. Random motility of cells is modeled by a Wiener Process *ξ*_*i*_ with expectation 〈*ξ*_*i*_(*t*)〉 = 0 and covariance 〈*ξ*_*i*_(*t*) · *ξ*_*j*_(*t*′)〉 = 2*Dδ*_*i*,*j*_ *δ*(*t*−*t*′), where *δ*_*i*,*j*_ denotes the Kronecker delta function, *δ*(*t*) the Dirac delta distribution (i.e. zero autocorrelation and no correlation between two different cells *i* and *j*). In the absence of any mechanical interactions, the noise term represents diffusion of the cells where *D* is the diffusion rate. The dynamics of the cell position is given as

3 $$\frac{{\rm{d}}{{\boldsymbol{x}}}_{i}}{{\rm{d}}t}=\sum _{k,k\ne i}{{\boldsymbol{F}}}_{i,k}+{\xi }_{i}\,.$$

This simple stochastic individual-based model and also the derived continuous version correctly reflect coordinated motility characterized by a high correlation in the velocities of adjacent cells. Simulations based on this model also show that the two processes, random motility and mechanical short-range interactions, are sufficient to allow gap closure for certain parameter sets, confirming previous studies.^35^ Random motility is hereby restricted by attractive forces between the cells and significant front propagation is only possible if the initial density of the cells is higher than the homeostatic cell density where *r* = *r*_*c*_. The main contribution to the front propagation is then the relaxation following tissue compression. However, speed dynamics of simulations based on Eq. (3) always show a monotonous decay (Fig. 3c). This is not surprising, as cell displacements are the result of repulsive forces, which constantly reduce when the cells spread. This feature of the model is in disagreement with the experimental data, as we observe for all experimental conditions (see Fig. 3a) a characteristic speed evolution profile exhibiting an initial increase in the average speed, a peak and a successive decline of average speeds. The simple model is thus not able to capture this qualitative behavior seen in the data. To achieve speed dynamics with initial acceleration and subsequent deceleration, and thus qualitative agreement between model and data, we extend the model by accounting for the fact that cells polarize in response to mechanical stretch and then migrate actively with a certain persistence time.^13^ We describe this active migration by a vector-valued velocity ${{\boldsymbol{x}}}_{i}^{\ast }=({x}_{i}^{\ast },{y}_{i}^{\ast })$, which is stimulated by forces between neighboring cells^36^ and decays exponentially with a characteristic constant *τ*. The extended model then reads:

4a $$\frac{{\rm{d}}{{\boldsymbol{x}}}_{i}}{{\rm{d}}t}=\sum _{k,k\ne i}{{\boldsymbol{F}}}_{i,k}+{{\boldsymbol{x}}}_{i}^{\ast }+{\xi }_{i}$$

4b $$\frac{{\rm{d}}{{\boldsymbol{x}}}_{i}^{\ast }}{{\rm{d}}t}=\frac{1}{\tau }({A}_{0}\sum _{k,k\ne i}{{\boldsymbol{F}}}_{i,k}-{{\boldsymbol{x}}}_{i}^{\ast }),$$

with *A*_0_ ≥ 0 determining how strongly active migration is stimulated through intracellular forces. For simulations the initial positioning of the cells resembled the situation in a scratch assay. Cells were seeded randomly in confined regions with *x*_*i*_ ∈ [−*L*:−*X*] for the left, and *x*_*i*_ ∈ [*X*:*L*] for the right monolayer. The middle of the domain, *x*_*i*_ ∈ (−*X*:*X*), remained cell-free. Removal of the insert is associated with mechanical stress and loss of contact inhibition, which stimulates polarization and migration of marginal cells towards the gap. This ‘leader cell (pioneer cell) behavior’ has been reported to be enhanced by treatment with growth factors.^19^ The initial stimulation of marginal cells was represented in the simulations by initial conditions ${x}_{i}^{\ast }=\theta \,$if *x*_*i*_ ∈ [−*X*−*ε*:−*X*] or ${x}_{i}^{\ast }=-\theta$ if *x*_*i*_ ∈ [*X*:*X *+ *ε*], with *ε* a small positive constant.

In the following we will present simulation results based on the model system Eq. (4). Please note that since all variables and parameters are dimensionless and scaled, direct quantitative comparison between simulations and experimental data is limited, but we will show that the model captures all experimentally observed features in a qualitative way. Quantitative comparison requires rescaling of the variables (see Supplementary Materials) and parameter fitting (see Discussion).

### The extended model reproduces in vitro speed dynamics of NSCLC cells

The extended model (Eq. 4) yields average speeds showing initial acceleration and subsequent deceleration (Fig. 3c, showing a simulation for a parameter set yielding straight fronts and effectively closed gaps). Acceleration (increase in the average speed of all cells) is a result of successive stimulation of active migration in increasing numbers of cells, as in the experiment (see Fig. 3b). In consequence, a wave of mechanical stimulation of ***x*** ^*^ sweeps from the front into the tissue, as shown in the kymograph (Fig. 3d). Since cell proliferation is not considered, front propagation is associated with cell density reduction, and an increase in the surface of individual cells. As the cells are adherent, the front propagation will eventually be antagonized by adhesive forces when the distance between neighboring cells becomes larger than *r*_*c*_. This interplay of forces explains the timing of the peak in the speed dynamics, also in the case when the gap does not close. Simulations based on the model are qualitatively in very good agreement with the experimental data (Fig. 3): (i) the speed evolution shows the characteristic acceleration–deceleration profile, (ii) the ratio between the maximal and initial speeds is similar between simulation (1.5 fold) and experiment (1.5–2 fold), (iii) the model reproduces the butterfly-shaped activation pattern seen in the data, (iv) and also the linear dynamics of front closure are captured. This agreement is very promising, especially since the model parameters were selected to yield straight fronts and effectively closed gaps (mimicking FCS treatment), but the model parameters were not specifically selected to achieve optimal agreement between simulation and data.

### The extended model reproduces different migratory behaviors

**Fig. 4:**
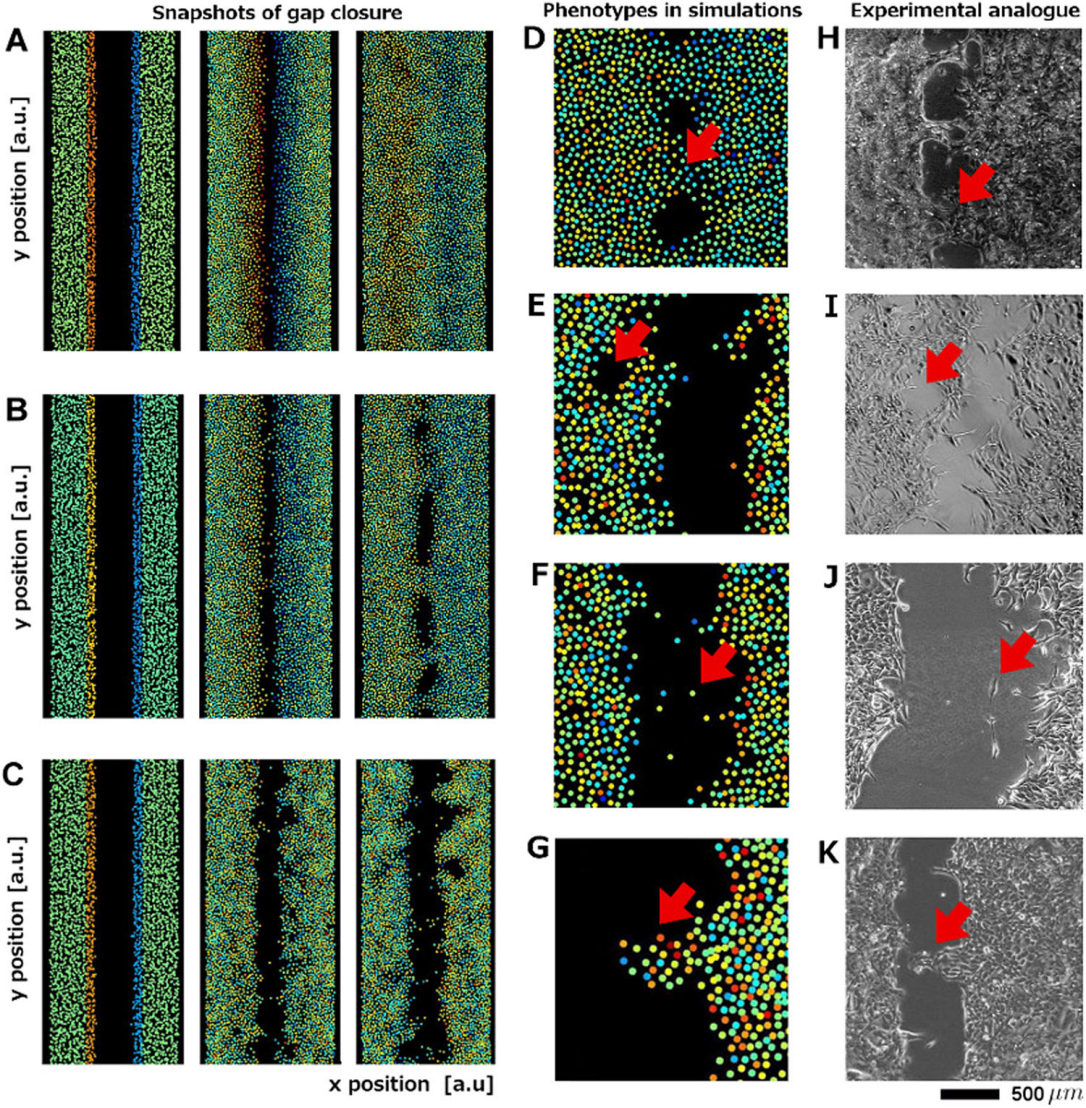
Different gap closure phenotypes observed in H1975 cells can be reproduced by the extended mathematical model using different parameter sets. (**a**) Straight fronts, here for strong initial stimulation of marginal cells and strong mechanotransduction. (**b**) Cellular bridges for more moderate mechanotransduction strength and weaker initial stimulation. (**c**) Undulating fronts for a smaller homeostatic cell size (resp. lower cell density). Parameters for (a)–(c) are listed in Table 2. The respective movies are provided as Supplementary Material. *Colors* encode cell velocity components perpendicular to the gap (*red*: fast movement in positive *x*-direction, *blue*: fast movement in the opposite direction). Panels (d)–(i) show further gap closure phenotypes reflected by the extended mathematical model as well as bright field pictures of their experimental analogs. Cellular bridges in simulation (**d**) and in unstimulated H1975 cells (**h**). Formation of small round lesions in simulation (**e**) and in H1975 cells treated with 100 ng/ml EGF (**i**). Detached cells in simulation (**f**) and for H1975 cells treated with 50 ng/ml IGF (**j**). The detachment and independent migration of individual cells or cell groups can occur naturally in the simulation since the connecting forces are of limited spatial range. (**g**) Finger-shapes in simulation, and (**k**) in an experiment involving untreated H1975 cells. Snapshots (**e**–**g**) originate from a simulation yielding undulating fronts with parameters listed in Table 2

Simulations based on (Eq. 4) also show that by varying the model parameters we are able to reproduce all observed phenotypes of gap closure, e.g. straight fronts, cellular bridges, and undulating fronts (Fig. 4a–c and Table 2). Straight fronts develop for instance when cells are seeded at a high density, where front propagation results from the relaxation of the cell surface. Stimulation of active migration by repulsive forces is hereby possible (*A*_0_ > 0, *τ* > 0), and in effect the speed dynamics show the characteristic peak. Alternatively, the fronts remain straight if the adhesion forces are weak (small *F*_0_ or small *σ*) or for strong initial activation combined with strong stimulation of ***x*** ^*^ (Fig. 4a). Cellular bridges (Fig. 4b) and undulating fronts (Fig. 4c) result for very similar parameter sets, and are indeed a similar phenomenon. Both cases are associated with the development of finger-like shapes (Fig. 4k). When the fingers reach the opposing front, cellular bridges are created (Fig. 4b, d, h). If the initial stimulation of the marginal cells *θ* is the same (as for the examples shown in Fig. 4), the cell density (i.e. *r*_*c*_) determines whether cellular bridges are formed or only finger-shapes. In our model, finger-like shapes develop without predefined leader cells. The fingers are a result of counter-acting forces, with active motion on one hand, restricted by adhesion on the other. The stochasticity of the model allows that some parts of the front surface engage in migration, while in other areas adhesion dominates. The emerging tip cells exhibit features often associated with a leader cell phenotype in the literature,^37, 38^ like a larger size than bulk cells. Also, the finger-shapes in our simulation display a density profile characterized by large cell–cell distances (respectively cell size) at the tip and decreasing cell–cell distances (smaller cells) towards the finger base (Fig. 4g). As the parameters for all models are the same, this shows that a leader cell-like phenotype can emerge solely because of the exposed position of the tip cell. In a previous study, it was found that the inhibition of Rho or ROCK does not inhibit cell motility, but prevents the formation of fingers.^38^ As Rho and ROCK are substantial components regulating cell polarization, this can be represented in our model by setting ***x*** ^*^ = 0, where no finger-shapes are possible.

**Table 2:** Model parameters leading to different phenotypes during gap closure

	

Varied parameters with respect to the default set leading to cellular bridges are shaded in *gray*

Apart from this, the model can capture further phenotypes often observed in the experiments: (a) In the case of the two monolayers forming cellular bridges we often observed a dynamic remodeling of these bridges: while new bridges developed, holes appeared in neighboring areas, where the gap had already closed. (b) In other cases small round lesions opened in the bulk tissue behind the propagating front (Fig. 4i). (c) Finally, while the studied NSCLC cell lines showed predominant collective migration, we also observed a detachment of individual cells from the front, which then migrated individually until they re-associated with the monolayer (Fig. 4j). All these features (a–c) are also inherent features of the extended model (Fig. 4d–g).

### The model can identify possible effects of chemical stimulation on mechanical properties of individual cells as well as the phenotype variability seen in experimental replicates

Simulations based on the extended model show that with sufficient stimulation of marginal cells (high |*θ*|), intact mechanotransduction *A*_0_ and not too strong adhesion *F*_0_ between the cells, the monolayer can effectively cover the artificial wound and achieve a permanent gap closure. Low initial stimulation and weak mechanotransduction, strong adhesion or mechanical stiffness yield non-motile or only weakly motile monolayers in the simulation, exhibiting finger structures and undulating fronts. In experiments, stimulation by FCS or single growth factors (EGF, IGF, and HGF) usually yields stronger front motility and effective gap closure with intact monolayers, indicating that cell-cell adhesion is not weakened by the treatment. Model simulations confirm that the resulting phenotypes (cellular bridges or complete gap closure involving straight fronts) can be obtained solely by increased initial stimulation of marginal cells and enhanced mechanotransduction (higher |*θ*| and *A*_0_, see Fig. 4a and Table 2), which is the mechanisms through which we believe transition between undulating fronts to cellular bridges (EGF/IGF/HGF treatment) or to straight fronts and closed gaps (FCS treatments) occurs. Alternatively, simulations show that gap closure can be achieved by disruption of adhesion (see Supplementary Materials).^25, 35^ Such situation can reflect the gap closure phenotype of cell lines in which the individual cells avoid cell–cell contacts. In an experimental setup involving non-adhering cells we would expect to find straight fronts and monotonously decaying speeds, while for adherent cells with intact mechanotransduction we expect to see the characteristic acceleration–deceleration speed profile.

On the other hand, simulations also show that enhanced motility, complete gap closure and straight fronts can be achieved by increasing the cell density, or, equivalently, by increasing the homeostatic cell size *r*_*c*_. To validate this finding experimentally we selected a set of experiments with strongly varying cell density. And indeed, in experiments with higher cell density we observed higher average speeds, straight fronts and fully closed gaps, while in replicates with lower cell density, speeds were slower, bridges/fingers and not fully closed gaps were common (see Supplementary Materials). A density effect is also visible in Fig. 1c. Here the right front exhibits a higher cell density at *t* = 0, and a larger number of finger structures at *t* = 14.5 h. It is likely that a locally high density contributes to the development of individual fingers and bridges.

When straight fronts are caused by high cell density and not by the absence of cell-cell adhesion, the speed dynamics can show a peak. Cell density can often not be precisely controlled in this experimental setup. Cell density variability is therefore likely to contribute to phenotype variability observed in replicates involving the same treatment (Table 1).

## Discussion

We have presented a mathematical model capable of reproducing many aspects of collective cell migration of NSCLC lung cancer cell lines under different treatment conditions. In this model we have identified a very small set of features that are sufficient and also necessary to produce all phenotypes and spatio-temporal dynamics observed in the data, as any further reduction or removal of features would render the model incapable of reproducing our experimental observations (see Supplementary Materials). For instance, while a model combining random motility and adhesion is sufficient to mimic gap closure of a collectively migrating cell system, the characteristic speed dynamics observed under all experimental conditions could only be reproduced when including active migration, stimulated through mechanical interaction between neighbors. Simulations based on the extended model, however, reflect many features seen in the data, such as the development of holes and finger-shapes, or the detachment of individual cells.

Many other modeling approaches for collective cell migration include a term for velocity alignment of adjacent cells^21, 24^ or specific design properties to ensure monolayer coherence, like lateral drag.^11^ These features are not explicitly included in our model. However, velocity alignment of neighboring cells emerges as a result of cell–cell adhesion,^34^ as well as through stimulation of directed motion by mechanical interaction. Also monolayer coherence emerges, to some degree, from the attractive part of the interaction potential. Specific model terms forcing complete coherence or lateral drag would prevent the formation of holes and the detachment of individual cells, which, however, are processes we wish to capture with the model. A further advantage of our model is that it is given in terms of plain mechanistic processes, where the effects of all model parameters can be associated with potential molecular regulators, e.g. effectors for mechanotransduction in collectively migrating cells,^39^ allowing for experimental validation (see Supplementary Materials), which is not possible for abstract terms like velocity alignment.

Our model assumes that all cells have an identical molecular setup, i.e. the model parameters are the same for every cell. This assumption is justified by the use of lung cancer cell lines that are expanded under standardized conditions and are only used at low passage number for the experiments. However, differences in the motility of individual cells can arise from the specific cell position, interaction structure, and the included term for random motility. Simulations based on the model show that further assumptions, e.g. cell parameter dependence on the spatial position, are not necessary to explain the obtained experimental observations.

In particular, simulations based on our model exhibit undulating front instabilities with finger-like shapes without the need to introduce leader cells with specific properties.^24^ We observed undulating front instabilities only in the extended model involving active migration stimulated by neighboring cells. In a previous study it was shown that lack of adhesion prevents undulating front instabilities,^25^ even though by construction of the model, the cells still exhibited velocity alignment despite missing adhesion.

Even though the model presented here is able to reflect many properties of collective cell migration following different treatments, simulations involving different parameter sets show that the phenotype alone (i.e. straight fronts vs. cellular bridges vs. undulating fronts and finger shapes) does not always allow to conclude about the effect of the treatment on the mechanical properties of individual cells. In particular gap closure involving straight fronts can be achieved in many ways: (a) when cells are seeded with high cell density, where repulsion is the major factor driving migration; or (b) when the cells tend towards larger cell sizes (due to growth or altered cell-substrate adhesion); (c) by low adhesion, as discussed above; or (d) when the initial stimulation of the marginal cells is strong and combined with a strong stimulation of active migration.

We have shown that the model captures all experimentally observed features of collective cell migration in the investigated cell lines in a qualitative way. The most important next step is the quantitative comparison, which requires rescaling of the model (see Supplementary Materials) and parameter fitting. Usually, parameter fitting is very tedious for individual-based models, since large numbers of simulations have to be conducted to obtain appropriate statistics. To avoid this, we are currently validating a mathematical approach made feasible by the simplicity of the model, which allows the direct estimation of model parameters based on single-cell tracking data. When successful, parameter estimation will provide an educated guess about which mechanical features or molecular regulators might differ between different cell lines, or be affected by a given treatment. Since the lateral migration assay is a standard and cost-inexpensive experimental technique, efficient parameter estimation will also allow the scanning and categorization of the function of a large number of different treatment approaches.

The model is presently describing cell migration on two-dimensional surfaces, which represents a popular experimental setup but not a realistic scenario. However, the model as given in Eq. (4) can describe cell migration in a three-dimensional domain without any extensions (using 3D coordinates, and velocities and forces given by vectors of length 3). Such 3D adaptation can provide a core model of developmental processes or tumor cell invasion in tumor spheroids. Since migration is not the only process important for cancer progression, further extensions might be required depending on the given experimental data and biological question addressed. Such additional features could be cell proliferation and apoptosis, interaction with the ECM, the response to external chemical or mechanical gradients, or the connection with internal signaling processes steering cell function.

What are the therapeutic implications of our study? Spatio-temporal analyses in vitro revealed that perturbation of signaling pathways relevant for the initiation and maintenance of a migration front phenotype (e.g. the EGF/EGFR pathway) only partly reduced tumor cell motility (Fig. 3a). These results are supported by previous data illustrating that different pathways cooperate in the regulation of NSCLC cell migration.^40^ In accordance with this, stimulations with distinct growth factors are not sufficient to induce a maximal migratory response (straight front migration) but instead account for a partial cellular response (e.g. bridge formation). These results suggest that individual perturbation approaches such as the inhibition of specific ligand/receptor combinations via highly selective kinase inhibitors can not completely shut down migration, which corresponds to the fact that NSCLC patients treated with single FDA-approved kinase inhibitors in most cases suffer from tumor remission and metastasis.^41^ Since collective cell migration and cluster formation favor the metastatic potential of epithelial tumor cells,^10^ our data suggest that combinations of inhbitors targeting a spectrum of motogenic factors could be more efficient strategies for the reduction of tumor cell dissemination.

In summary, we here demonstrate that an integrative approach combining time-resolved and quantitative measurements of tumor cells with and without signaling pathway stimulations/perturbations can be used for the generation of a mathematical model, which can be used to study possible effects of motogenic stimulation or inhibition on mechanical cell–cell interaction and motility. Importantly, only a small set of specific mechanical parameters are sufficient to describe the spatio-temporal and phenotypic behavior of highly motile NSCLC cells, illustrating that these features in combination with the respective extracellular input information define the mode of tumor cell migration.

## Methods

### Cell lines, cell culture, cell stimulation, and perturbation

Human lung adenocarcinoma cell lines H1975 and H1650 were purchased from ATCC (CRL-5908 and 5883) and were cultivated in Dulbecco’s modified Eagle’s Medium (DMEM, Lonza) supplemented with 10% FCS (Gibco), 100 μg/ml streptomycin (Gibco) and 100 U/ml penicillin (Gibco). The adherent cells were expanded on 10 cm dishes in a humid atmosphere containing 5% CO_2_ at a temperature of 37 °C and were only used at a low passage number for the experiments. To decrease background activation of signaling pathways by FCS, the cells were growth factor depleted. To this end, the medium was aspirated and the cells were washed at least two times with PBS. Subsequently, medium containing 1% BSA (growth factor depletion medium) was added and the cells were incubated overnight. The following day, cells were stimulated with different concentrations of (IGF-1) and/or EGF diluted in growth factor depletion medium (10 ng/ml). For the perturbation of signaling, the cells were treated with the EGFR inhibitor erlotinib (iEGFR) using different concentrations (50 and 100 nM).

### Lateral migration assay and live cell imaging data acquisition

For migration assays cells were seeded in IBIDI inserts (IBIDI, Martinsried, Germany) in a 24-well plate (2 × 10^4^ cells in each insert chamber) and left to attach overnight in the incubator. The inserts were carefully peeled off, resulting in a gap of 500 μm between the populations. The wells were washed and subsequently filled with 500 μl of the respective medium. Cell nuclei were stained with NucBlue Live ReadyProbes (Life Technologies, Darmstadt, Germany) according to the manufacturers’ protocol. Live-cell imaging was conducted using an Olympus Cell^R^ Live Cell Imaging System with an IX81 motorized inverted Microscope and a Hamamatsu ORCA-R2 camera, fitted with a climate chamber. Images of lateral migration over 72 h were acquired using the Olympus excellence RT software with four times magnification in both phase contrast and at 460 nm channels in intervals of 90 min between pictures. Each gap was covered by a vertical line of five pictures with an overlap of ~20%. Image stacks were stitched using the stitch grid of images plugin in FIJI, with automated computation of image overlap.

Only experiments with homogenous distribution of cells were analyzed in this study to guarantee identical migration conditions for all within the chambers and to avoid position-dependent biased results.

### PIV analysis

Following contrast enhancement in ImageJ,^42^ we applied particle image velocimetry ^32^ using MatPIV,^43^ which computes cross-correlations between small interrogation areas on subsequent images. We used interrogation areas of 64 × 64 pixels with 50% overlap, and fitted a 2D Gaussian to determine cell displacements with subpixel resolution. Spurious vectors were removed by applying local, global, and signal-to-noise-ratio filters. Speeds (absolute velocities, |v|) were computed by dividing the displacements by the inter-frame time interval. Time-resolved speed dynamics were obtained by averaging the speed over the entire frame. To generate kymographs each image was divided into rectangular boxes and for each time-point the respective quantity (e.g. speed, *x*-component of the velocity, etc.) was averaged over the entire box. For PIV-derived pathlines we generated a mask of the initial gap area by manual segmentation of the first movie frame. Then 5000 random locations in the no-gap area were chosen as starting points for the trajectories. New positions were then computed from the PIV displacement field.

### Model implementation

The model is given in terms of non-dimensional variables, which were derived as described previously.^34^ Simulations of the individual-based models (Eqs 1–4) were computed using the Euler-Maruyama method implemented in C++.^44^ Results were imported into MATLAB for visualization. For both models we simulated the collective migration of approximately 5000 cells, which corresponds to the cell number in the experimental setup.

## Electronic supplementary material

Supplementary Information

Supplementary Movies

## References

[1] Bielefeld KA, Amini-Nik S, Alman BA (2013). Cutaneous wound healing: recruiting developmental pathways for regeneration. Cell. Mol. Life Sci..

[2] Fan X, Zhang X, Wang H, Jin B (2014). Reevaluation of survival and prognostic factors in pathologic stage I lung adenocarcinoma by the new 2009 TNM classification. Tumour Biol..

[3] Yu HA (2015). Differences in the survival of patients with recurrent versus de novo metastatic KRAS-mutant and EGFR-mutant lung adenocarcinomas. Cancer.

[4] Lee JG (2008). Number of metastatic lymph nodes in resected non-small cell lung cancer predicts patient survival. Ann. Thorac. Surg..

[5] Fukui T, Mori S, Yokoi K, Mitsudomi T (2006). Significance of the number of positive lymph nodes in resected non-small cell lung cancer. J. Thorac. Oncol..

[6] Warth A (2015). Prognostic impact of intra-alveolar tumor spread in pulmonary adenocarcinoma. Am. J. Surg. Pathol..

[7] Clark AG, Vignjevic DM (2015). Modes of cancer cell invasion and the role of the microenvironment. Curr. Opin. Cell Biol..

[8] Sahai E, Marshall CJ (2003). Differing modes of tumour cell invasion have distinct requirements for Rho/ROCK signalling and extracellular proteolysis. Nat. Cell Biol..

[9] Sanz-Moreno V (2008). Rac activation and inactivation control plasticity of tumor cell movement. Cell.

[10] Friedl P, Gilmour D (2009). Collective cell migration in morphogenesis, regeneration and cancer. Nat. Rev. Mol. Cell Biol..

[11] Vitorino P, Hammer M, Kim J, Meyer T (2011). A steering model of endothelial sheet migration recapitulates monolayer integrity and directed collective migration. Mol. Cell Biol..

[12] Serra-Picamal X (2012). Mechanical waves during tissue expansion. Nat. Phys..

[13] Kolega J (1986). Effects of mechanical tension on protrusive activity and microfilament and intermediate filament organization in an epidermal epithelium moving in culture. J. Cell Biol..

[14] Simmers MB, Pryor AW, Blackman BR (2007). Arterial shear stress regulates endothelial cell-directed migration, polarity, and morphology in confluent monolayers. Am. J. Physiol. Heart Circ. Physiol..

[15] Farooqui R, Fenteany G (2005). Multiple rows of cells behind an epithelial wound edge extend cryptic lamellipodia to collectively drive cell-sheet movement. J. Cell Sci..

[16] Fenteany G, Janmey PA, Stossel TP (2000). Signaling pathways and cell mechanics involved in wound closure by epithelial cell sheets. Curr. Biol..

[17] Trepat X (2009). Physical forces during collective cell migration. Nat. Phys..

[18] Altan ZM, Fenteany G (2004). c-Jun N-terminal kinase regulates lamellipodial protrusion and cell sheet migration during epithelial wound closure by a gene expression-independent mechanism. Biochem. Biophys. Res. Commun..

[19] Vitorino P, Meyer T (2008). Modular control of endothelial sheet migration. Genes Dev..

[20] Vicsek T, Czirok A, Ben-Jacob E, Cohen II, Shochet O (1995). Novel type of phase transition in a system of self-driven particles. Phys. Rev. Lett..

[21] Szabo B (2006). Phase transition in the collective migration of tissue cells: experiment and model. Phys. Rev. E.

[22] Mehes E, Vicsek T (2014). Collective motion of cells: from experiments to models. Integr. Biol..

[23] Kabla AJ (2012). Collective cell migration: leadership, invasion and segregation. J. R. Soc. Interface.

[24] Sepulveda N (2013). Collective cell motion in an epithelial sheet can be quantitatively described by a stochastic interacting particle model. PLoS Comput. Biol..

[25] Basan M, Elgeti J, Hannezo E, Rappel WJ, Levine H (2013). Alignment of cellular motility forces with tissue flow as a mechanism for efficient wound healing. Proc. Natl. Acad. Sci. USA.

[26] Swat MH (2012). Multi-scale modeling of tissues using CompuCell3D. Methods Cell. Biol..

[27] Starruss J, de Back W, Brusch L, Deutsch A (2014). Morpheus: a user-friendly modeling environment for multiscale and multicellular systems biology. Bioinformatics.

[28] Fletcher AG, Osterfield M, Baker RE, Shvartsman SY (2014). Vertex models of epithelial morphogenesis. Biophys. J..

[29] Lober J, Ziebert F, Aranson IS (2015). Collisions of deformable cells lead to collective migration. Sci. Rep..

[30] Mirams GR (2013). Chaste: an open source C++ library for computational physiology and biology. PLoS Comput. Biol..

[31] Scagliotti GV, Masiero P, Pozzi E (1995). Biological prognostic factors in non-small cell lung cancer. Lung Cancer.

[32] Raffel, M., Willert C. E., Wereley S. and Kompenhans J. *Particle image velocimetry—a practical guide* (Springer, 1998).

[33] Petitjean L (2010). Velocity fields in a collectively migrating epithelium. Biophys. J..

[34] Middleton AM, Fleck C, Grima R (2014). A continuum approximation to an off-lattice individual-cell based model of cell migration and adhesion. J. Theor. Biol..

[35] Bindschadler M, McGrath JL (2007). Sheet migration by wounded monolayers as an emergent property of single-cell dynamics. J. Cell Sci..

[36] Serra-Picamal X (2012). Mechanical waves during tissue expansion. Nat. Phys..

[37] Poujade M (2007). Collective migration of an epithelial monolayer in response to a model wound. Proc. Natl Acad. Sci. USA.

[38] Reffay M (2014). Interplay of RhoA and mechanical forces in collective cell migration driven by leader cells. Nat. Cell Biol..

[39] Das T (2015). A molecular mechanotransduction pathway regulates collective migration of epithelial cells. Nat. Cell Biol..

[40] Johnson H, Lescarbeau RS, Gutierrez JA, White FM (2013). Phosphotyrosine profiling of NSCLC cells in response to EGF and HGF reveals network specific mediators of invasion. J. Proteome Res..

[41] Omuro AM (2005). High incidence of disease recurrence in the brain and leptomeninges in patients with nonsmall cell lung carcinoma after response to gefitinib. Cancer.

[42] Schneider CA, Rasband WS, Eliceiri KW (2012). NIH Image to ImageJ: 25 years of image analysis. Nat. Methods.

[43] Sveen, J. K. An introduction to MatPIV v.1.6.1. Dept of Math University of Oslo, mechanics and applied mathematics, 0809-4403 (2004).

[44] Platen E (1999). An introduction to numerical methods for stochastic differential equations. Acta Numer..

