# Supplementary material for: An individual-based model for collective cancer cell migration explains speed dynamics and phenotype variability in response to growth factors
Source: NPJ Syst Biol Appl. 2017 Mar 3;3:5. doi: 10.1038/s41540-017-0006-3 (PMC5460121; doi:10.1038/s41540-017-0006-3)
Supplement: Supplementary file 1 — Supplementary Information [file 41540_2017_6_MOESM1_ESM.docx]

An individual-based model for collective cancer cell migration explains speed dynamics and phenotype variability in response to growth factors

Damian Stichel, Alistair M. Middleton, Benedikt F. Müller, Sofia Depner, Ursula Klingmüller, Kai Breuhahn, Franziska Matthäus

Supplementary Material

1. **The different front phenotypes are due to cell migration and do not require proliferation**

This is supported by a data set testing for the effect of mitomycin on the migration behavior of H1975 cells. Cells were either untreated or subjected to treatment with full medium (FCS). The experiments were repeated with additional inhibition of cell proliferation using mitomycin-C. Cell migration was monitored over 68 hours. Independent from mitomycin-C treatment, starved cells showed very little to no motility and did not close the gap. Also independent from mitomycin-C treatment, stimulation with FCS induced straight front behavior and a fully closed gap (Table S1).

**Table S1**

**Results for an experiment involving H1975 cells without stimulation or stimulation using FCS.** In half of the experiments proliferation was inhibited by mitomycin-C. The gap closing phenotype (low motility/no gap closure vs. straight front phenotype with effective gap closure) depended only on the stimulation, not on cell proliferation.

|  | Without mitomycin-C | With mitomycin-C |
| --- | --- | --- |
| Control (starvation) | Low motility (5/5) | Low motility (5/5) |
| FCS | Close gap / straight front (5/5) | Close gap / straight front (5/5) |

In an experiment involving stimulation by FCS, EGF we observe the three phenotypes – finger-like structures, cellular bridges, and straight fronts also when proliferation is inhibited by mitomycin-C (Figure S1).


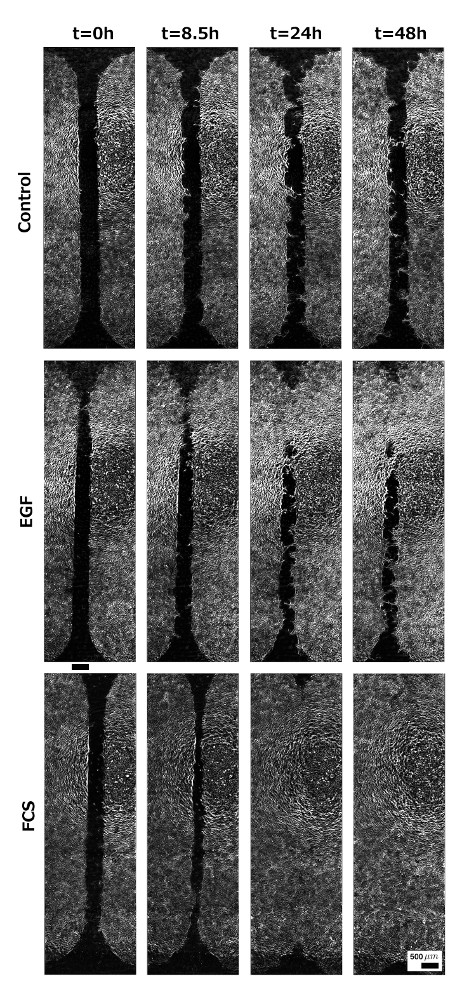


**Figure S1:**

**Different front phenotypes occur for different treatments also when proliferation is inhibited by mitomycin-C.** Snapshots (phase contrast images) are shown for experiments involving no stimulation (top) – finger structures, EGF stimulation (middle) with one large cellular bridge, and FCS stimulation (bottom) exhibiting straight front closure, for four different time points (0h, 8.5h, 24h, 48h). Contrast was enhanced between cell-containing and cell-free areas by background removal using the image processing software ImageJ.

**2. High cell density promotes efficient gap closure and straight front migration**

Experiments carried out under the same conditions (same cell line and treatment) did not always show the same gap closure dynamics or phenotypes. In our experiments, this variability seemed to be partially explained by the different cell densities. To confirm our hypothesis we used 15 replicates of an experiment involving H1975 cells treated with 40 ng/ml HGF. These movies were of sufficient quality to allow the detection of stained cell nuclei, and thus cell counting, on the first frame. The first frame of each movie was preprocessed in ImageJ, involving the selection of an image segment of uniform size (0.16x0.29cm), contrast enhancement and smoothing. Nuclei detection was carried out using u-track [1], a software package for particle detection and tracking implemented in MATLAB. Speeds averaged over 24 hours and over the entire domain were computed from PIV displacement fields. Cell density and average speeds showed a positive correlation with coefficient 0.47. Based on the cell density the experiments could be split well into two groups showing also different average speeds (Figure S2).


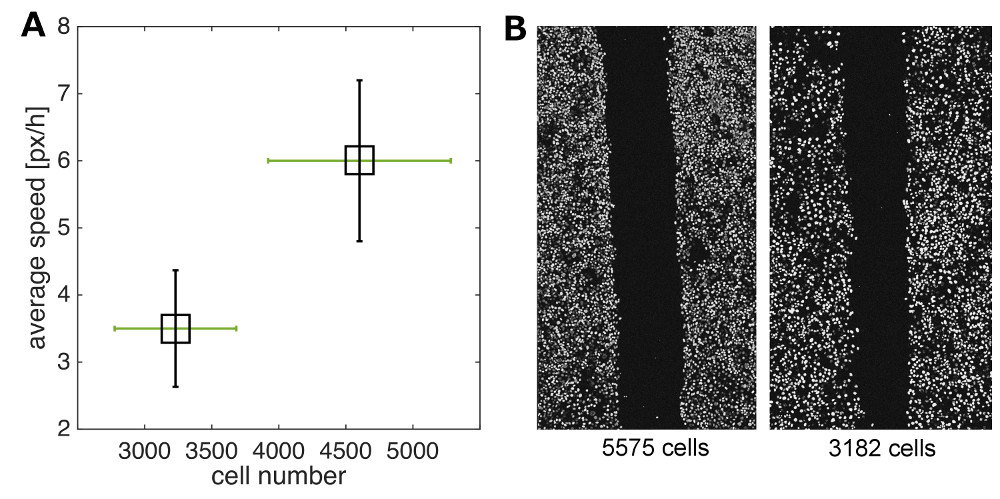


**Figure S2:**

**Comparison of average speeds measured by PIV for experiments with different cell densities.** Cell numbers were estimated using the software package u-track implemented in MATLAB, using only cell nucleus detection for the first frames of each movie. **A** Experiments were grouped into one group with low (7 replicates) and another with high cell density (8 replicates). Both groups clearly differed also in the average speeds. Error bars indicate standard deviation of average speeds and cell number. Speeds are given in pixel lengths (1.29$\mu m)$ per hour. **B** Shown are the first frames of an exemplary movie with high cell density (left) and another experiment with low cell density (right).

In the same data set we observed that the gap was completely closed after 24 hours in 7 out of 8 experiments with high cell density (on average after 11.9 hours), in every case connected with straight front closure. In contrast, the gap closed in only 2 out of 7 experiments with low cell density (after 22 and 23 hours respectively). Here we observed straight fronts in 3 experiments, the formation of cellular bridges in 3 experiments, and undulating fronts / finger structures in one experiment.


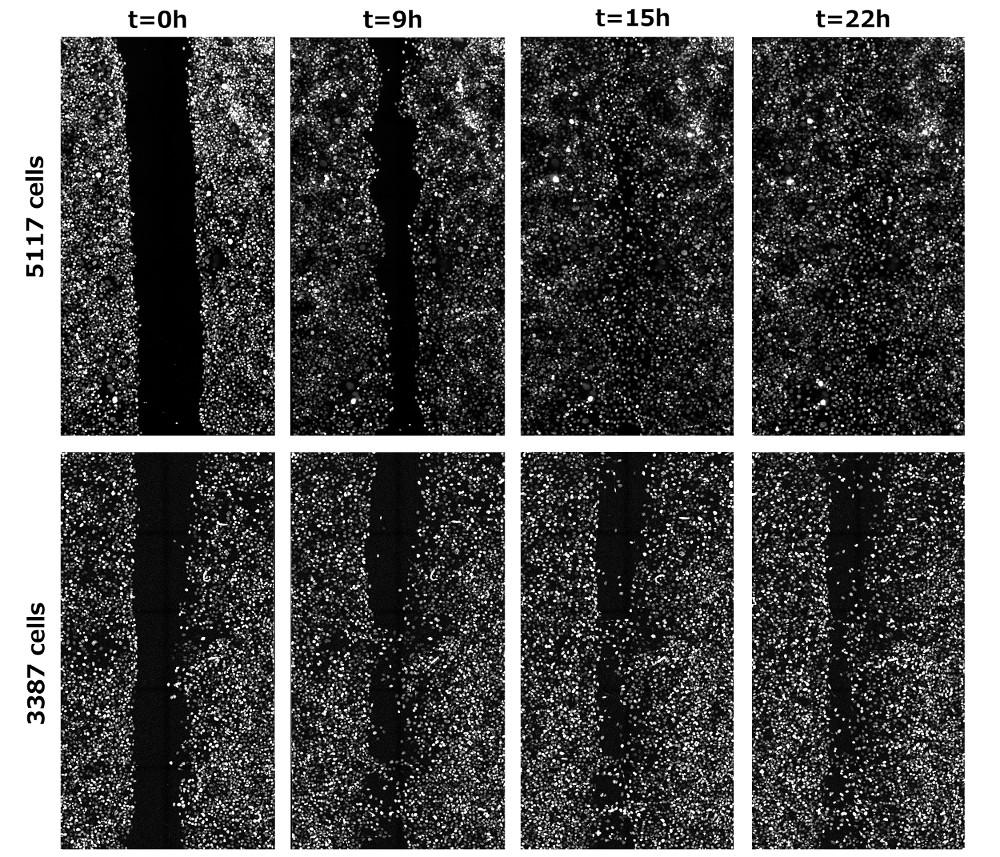


**Figure S3:**

**Snapshots of two experiments with different cell density**. Snapshots were taken at 0h, 9h, 15h and 22h. In the experiment with higher cell density (top, 5117 cells), the gap closed with a relatively straight front after 15 hours, while for the experiment with low cell density (bottom, 3387 cells) several bridges form. Here the gap is still visible after 22 hours.

1. **Activation of migration is initiated at the front and extends into the tissue**

In the document we stated that active migration is usually initiated at the tissue boundary and then extends into the tissue when more and more layers of submarginal cells are stimulated to engage in collective migration. Figure S4 shows the kymographs for the three phenotypes shown in Figure 1. Here, the color-coding was adjusted for the range of velocities seen in the located (and less strongly backpropagating) activation wave in the experiment involving IGF stimulation.


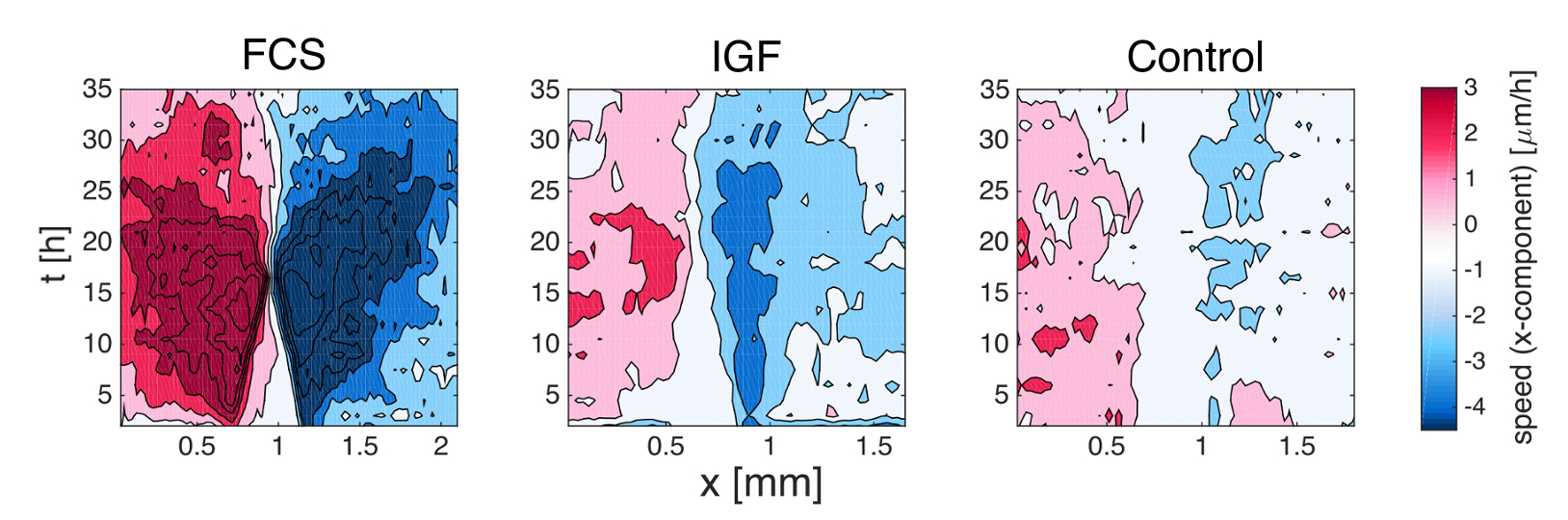


**Figure S4: Velocity kymographs (x-component of velocity) for the three different phenotypes.** Left: Migration is initiated at the front and propagates into the tissue for FCS treatment. Middle: Activation patterns of migration are more restricted to the boundary for IGF treatment. Right: Migration is weak and patterns are more distributed for starved cells.

1. **Simulations yield straight fronts through different mechanisms**

Figure 4 in the main document shows different migration phenotypes when the model parameters are varied. As described in the main document, the straight front phenotype can be obtained through different mechanisms. As shown in Figure 4, straight front closure can be achieved by high initial stimulation of the marginal cells (large $\theta$) in addition to strong mechanotransduction (large $A_{0}$). Alternatively, we report that straight fronts and complete gap closure also results from weak adhesion or high cell density. Snapshots of simulations for both scenarios are shown below. Low adhesion can be obtained in the model in different ways. One option would be to decrease the cell stiffness $a$. Lower stiffness yields weaker adhesion, but in the same instance also weaker repulsion. An alternative is to reduce $\sigma$, i.e. the cell-cell interaction radius. Two cells experience a repulsive force for a distance smaller than their natural size, given by the parameter $r_{c}$. Adhesion plays a role for distances between $r_{c}$ and $\sigma r_{c}$. In our default parameter set we have $\sigma=2$, hence, cells do not interact if they are further apart than $2r_{c}$. Adhesion is reduced by setting $1\leq\sigma<2$. For $\sigma<1$ adhesion is eliminated, but also repulsion is reduced. For $\sigma=0$ cells do not interact mechanically. To obtain Figure S5 (A), we decreased the interaction radius to $\sigma=1.26$ while keeping all further parameters unchanged (with respect to the default parameters yielding cellular bridges).

Cell density can be varied indirectly through the homeostatic cell size $r_{c}$. In the simulation shown in Figure S5 (B) we chose the more direct way of increasing the number of simulated cells by 1.5-fold (7200 instead of 4800). All further parameters are unchanged, see Table S2.


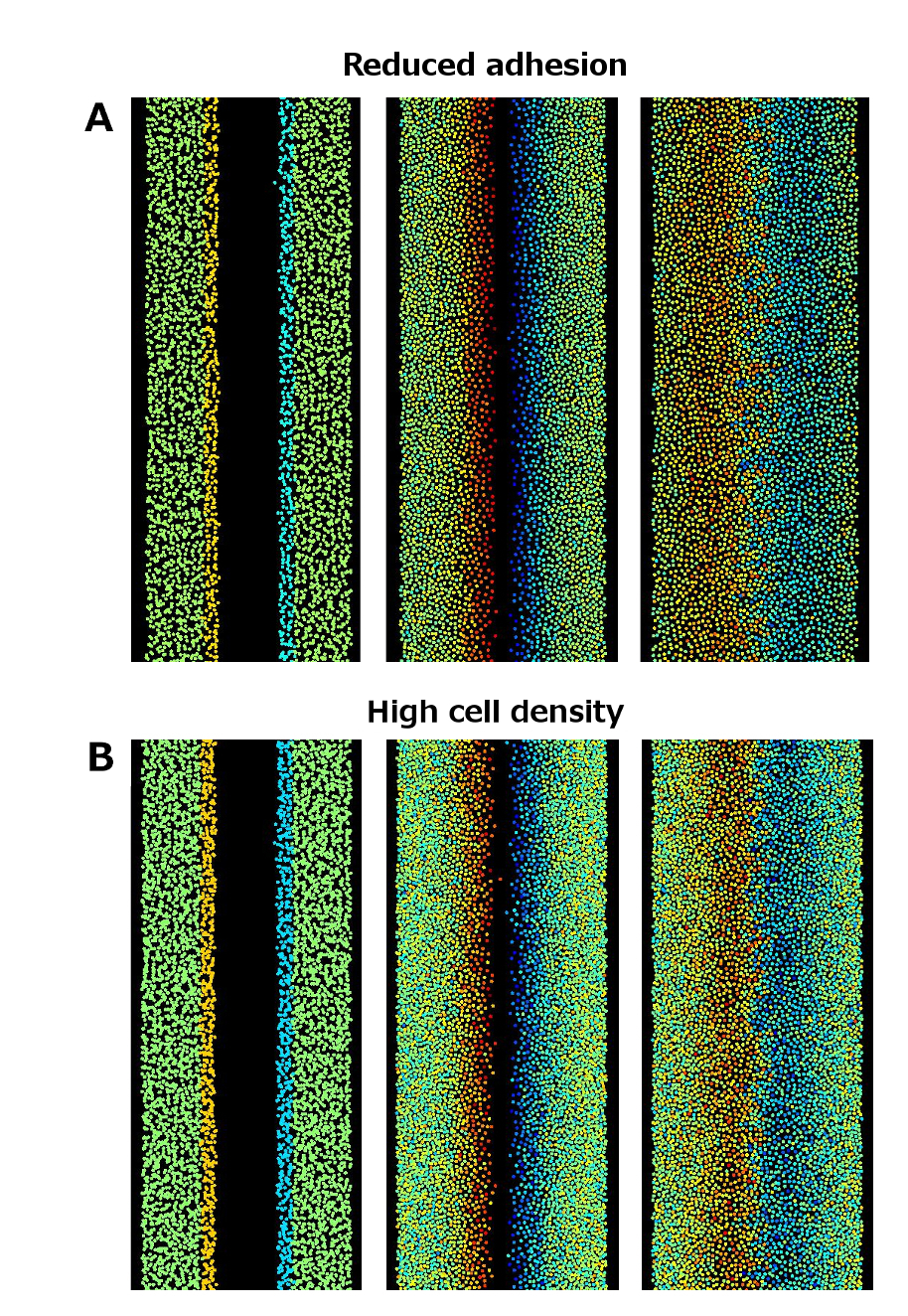


**Figure S5:** **Snapshots of gap closure in simulations with reduced adhesion or higher cell density.** For parameter values see Table S2 and the text above. For both cases we observe straight fronts and complete closure.

**Table S2: Parameter set yielding straight fronts and complete gap closure through reduced adhesion or higher cell density, shown in Figure S5.**

| **Parameter** | **Default parameters**  **yielding cellular bridges** | **Reduced adhesion**  **(Figure S5 A)** | **Higher cell density**  **(Figure S5 B)** |
| --- | --- | --- | --- |
| $\boldsymbol{D}$ | 0.01 | 0.01 | 0.01 |
| $\boldsymbol{F}_{\boldsymbol{0}}$ | 1 | 1 | 1 |
| $\boldsymbol{a}$ | 1.818 | 1.818 | 1.818 |
| $\boldsymbol{\tau}$ | 3.1623 | 3.1623 | 3.1623 |
| $\boldsymbol{\sigma}$ | 2 | 1.26 | 2 |
| $\boldsymbol{r}_{\boldsymbol{c}}$ | 0.0399 | 0.0399 | 0.0399 |
| $\boldsymbol{A}_{\boldsymbol{0}}$ | 100 | 100 | 100 |
| $\boldsymbol{\theta}$ | 1 | 1 | 1 |
| **Cells** | 4800 | 4800 | 7200 |

1. **The set of included features is sufficient and necessary to reproduce experimentally observed phenomena**

As shown in the main document, the extended model is sufficient to reproduce the observed experimental features, including the evolution of the average speeds, the spatio-temporal velocity distribution, and the different front closure phenotypes (straight fronts, cellular bridges, finger-structures). To underscore that these features are both necessary and sufficient to reproduce the experimental observations we will discuss possible model reductions and their effect on the simulation results.

1. No mechanotransduction ($A_{0}=0$)

This version represents the simple version of the model (Eq. 3) which is not able to produce the acceleration deceleration profile in the speed dynamics. Without mechanotransduction we also do not obtain finger-structures.

1. Mechanotransduction, but no friction (infinite persistence) can be achieved by removing the second term ($-x_{i}^{*}$ in the rhs of Equation (4b)). Without friction the model can generate artifacts since actively migrating single cells or cell groups would never stop. In our setup simulations without friction gave reasonable results as long as the cells remained connected in the tissue, and persistent migration was eventually stopped by the opposing front.
2. No random motility ($D=0$):

Without stochasticity the distances between neighboring cells are determined solely by the local forces and turn out to be very homogeneous. Stochastic differences in local speeds or cell densities do not occur. As a result the fronts remain straight, finger-structures do not occur (Figure S6 A).

1. No adhesion ($\sigma=1$):

Also without adhesion finger structures are not possible. (Figure S5 A) Furthermore, cell-cell adhesion is the biological requirement for mechanotransduction. When adhesion is removed from the model, it is implausible that cells are stimulated by pulling forces into active motion. We would thus also have to set $A_{0}=0$, disabling peaked speed evolution profiles.

1. No cutoff ($\sigma$large or infinite):

Mechanical cell-cell interaction (repulsion and adhesion) requires direct cell-cell contact. It is therefore biologically implausible for cells to interact mechanically over many cell diameters. In addition, this implies long-range attraction, as it is mainly the attractive (adhesive) part which is extended over longer distances. Long-distance attraction leads to cell aggregation, known for systems where cells produce a diffusible long-range chemoattractands. Also in our model we observe aggregation when the cutoff distance is increased (Figure S6B).

1. No repulsion (can be achieved through high elasticity, e.g. ($a=1$)):

Repulsion accounts for the fact that cells occupy a certain area and are not arbitrarily compressible. Apart from the fact that it is biologically implausible, removal of repulsion leads to numerical artifacts if adhesion is still present. In particular, if adhesion (attraction) is still present, local cell aggregates will form (Figure S6 C), which is not seen in the data.

In summary the model includes a very small set of features (adhesion, repulsion, random motility, mechanotransduction). The simpler model including only adhesion, repulsion and random motility was not able to capture all phenomena seen in the data, especially the peaked speed evolution profile, and closing fronts including finger structures and cellular bridges.


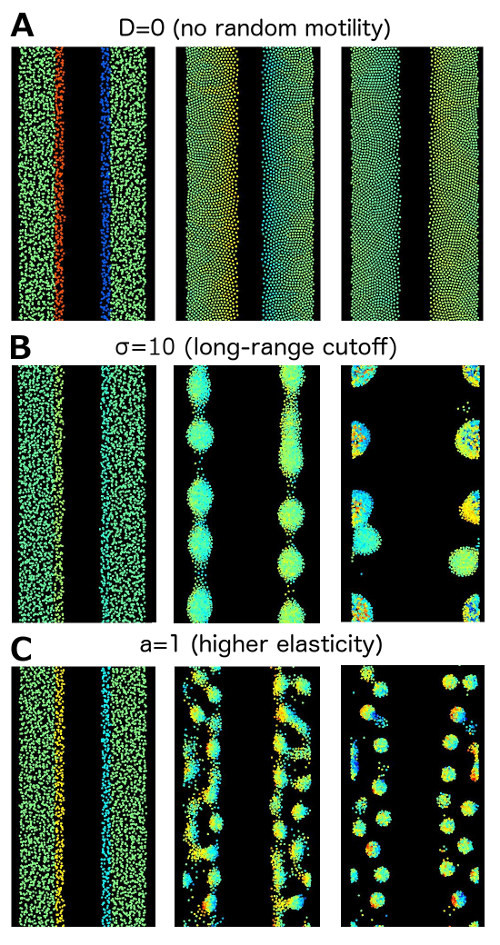


**Figure S6:** **Simulation snapshots for default parameters (Table S2) with small modifications corresponding to model reductions.** A) Without random motility (D=0) the model is not able to generate undulating fronts / finger structures. In this simulation the fronts move inward and then retract. B) If cell-cell interaction (particularly adhesion) over many cell diameters is allowed (large or no cutoff), this leads to long-range attraction and the formation of cell aggregates. C) Similar aggregates happen if repulsion is removed or reduced (here: low a). Adhesion then dominates and extends over several cell diameters because distances between individual cells can be low. This also leads to cell aggregates.

1. **Motivation for using PIV analysis**

Most image sequences analyzed for this study were originally obtained with the aim of quantifying the degree of gap closure on two snapshots at defined time points. Therefore, the image section was chosen such that the entire gap was contained, as well as a large number of cells. This choice, however, brought upon difficulties in nuclei segmentation. Attempts on single-cell tracking did not yield satisfactory results. Most of the problems with single-cell tracking became clear after visual inspection of the tracks, but could not be resolved due to the insufficient image quality.

We therefore chose to use an implementation of PIV, which does not allow the tracking of individual cells, and therefore does not provide the information on the history of the individual cell position or detail of the dynamics of the cell-cell interaction structure. However, PIV can deal with cell clusters, where individual cells can’t be segmented, and is also applicable for phase contrast images. Furthermore, PIV is robust and computationally effective. Using PIV we were able to quantitatively analyze the large amount of movies collected by our experimental partners (all in all 1500 throughout the last ~4 years).

1. **From qualitative to quantitative modeling**

The model is given in dimensionless variables by scaling with respect to the domain size and the macroscopic diffusion coefficient. For a quantitative comparison between model and data, variables and parameters of the model have to be rescaled as follows:

$\tilde{x}=x\cdot L$,

$\tilde{t}=t\cdot L^{2}/D$,

$\tilde{x}^{*}=x^{*}\cdot L$,

$\tilde{F}_{0}=F_{0}\cdot D/L$,

$\tilde{r}_{c}=r_{c}\cdot L$,

$\tilde{a}=a/r_{c}$,

$\tilde{\tau}=\tau\cdot L^{2}/D$,

$\tilde{A}_{0}=A_{0}\cdot D/L$,

$\tilde{\theta}=\theta\cdot L$,

whereby the tilde denotes the variables/parameters with units, $L$ denotes the domain size (x-axis), hier e.g. 2mm and $D$ the macroscopic diffusion coefficient, which has to be adjusted to feasible magnitudes, e.g. using published values (if possible). The model parameter not listed in the rescaling rules is the factor $\sigma$, which remains unchanged.

1. **Possible experimental approaches for model validation**

The model can be used to predict how the migration behavior of the monolayer may change when mechanical properties of the individual cells are varied. Different experimental approaches can be used to validate these predictions:

*Change of microenvironment*

To experimentally confirm if the model parameter mechanotransduction can affect cellular migration, cells could be seeded in dishes with variable surface elasticity and stiffness. For example, fibronectin-coated acrylamide hydrogels of varying stiffness ranging from 0.7 to 40 kPa, that represent correlates for physiological tissue elasticity [2]. In this context, stiff hydrogels range from 15-40 kPa while soft matrices range from 0.7 to 1 kPa).

In order to study adhesion processes, the culture of cells can be performed on micromolded elastomeric microposts that influence surface rigidity and the formation of focal adhesions (rigidity ranging from 1.3 to 1,550 nM µm^-1^) [3]. Changing rigidity directly regulates the spatial organization of focal adhesions and the induction of focal adhesion stress (ratio of traction force to corresponding focal adhesion area). Thus, these materials represent suitable surfaces for the analysis of focal adhesions on cell migration.

To test for changes in elasticity of single cells, changes in ECM stiffness and rigidity can be used as they impose different degrees of cell elasticity and spreading. To test if cell spreading is sufficient to regulate the local cell migration behaviour, defined micro-patterned fibronectin islands are used, which cause different degrees of cell spreading depending on the available surface area [4]. Using single cell microscopy, this approach could clarify if the cell spreading can affect cell mobility.

*Genetic approach*

More specific experimental approaches could include the genetic manipulation of cell lines including stable inhibition (shRNA or CRISPR/Cas9) or vector-based overexpression of genes directly involved in the formation of focal adhesions and elasticity (e.g. integrins) and mechanostransduction (e.g. focal adhesion complex components) followed by time-resolved analysis of cell migration.

*Chemical activators or inhibitors*

Chemical inhibitors targeting adhesion molecules like integrins [5], cadherins [6] or FAK [7], are presently used also in the treatment of cancers. FAK inhibition also affects levels of Merlin [8]. Also small compound inhibitors affecting regulators of cell elasticity are known [9]. Chemical inhibitors, however, often suffer from the lack of specificity. Furthermore, many of the regulators are involved in different cell functions. Merlin/NF2, for instance, does not only affect polarization in cell migration, but also is a central component in the Hippo/YAP signaling pathway regulating density dependence of growth and motility.

Lastly, drug treatment could be combined with further data collection (e.g. transcriptomics and/or proteomics) to allow the inference of affected cell functions or pathways. For example we intend to analyse cells treated with motogenic growth factors by time-resolved expression profiling followed by gene enrichment analysis to validate the model and the parameter estimation method.

**References**

[1] K. Jaqaman, D. Loerke, M. Mettlen, H. Kuwata, Sergio Grinstein, S.L. Schmid and G. Danuser, Robust single-particle tracking in live-cell time-lapse sequences, *Nature Methods* 5:695-702, 2008.

[2] A.J. Engler, S. Sen, H.L. Sweeney and D.E. Discher, Matrix elasticity directs stem cell lineage specification, Cell 126(4): 677-89, 2006.

[3] J. Fu, Y.K. Wang, M.T. Yang, R.A. Desai, X. Yu, Z. Liu and C.S. Chen, Mechanical regulation of cell function with geometrically modulated elastomeric substrates, Nature Methods 7(9):733-6, 2010.

[4] C.S. Chen, M. Mrksich, S. Huang, G.M. Whitesides, and D.E. Ingber, Geometric control of cell life and death 276(5317):1425-8, 1997.

[5] K. Ley, J. Rivera-Nieves, W.J. Sandborn and S. Shattil, Integrin-based therapeutics: biological basis, clinical use and new drugs, Nature Reviews Drug Discovery 15, 173–183, 2016.

[6] H. Li, D.K. Price, W.D. Figg, ADH1, an N-cadherin inhibitor, evaluated in preclinical models of angiogenesis and androgen-independent prostate cancer, Anticancer Drugs 18(5):563-8, 2007.

[7] K.B. Dunn, M. Heffler and V. Golubovskaya, Evolving Therapies and FAK Inhibitors for the Treatment of Cancer, Anticancer Agents Med Chem. 10(10): 722–734, 2010

[8] R.A. Stahel, W. Weder, E. Felley-Bosco, U. Petrausch, A. Curioni-Fontecedro, I. Schmitt-Opitz and S. Peters, Searching for targets for the systemic therapy of mesothelioma, Annals of Oncology 26 (8): 1649-1660, 2015.

[9] T.T. Bonello, M. Janco, J. Hook, A. Byun, M. Appaduray, I. Dedova, S. Hitchcock-DeGregori, E.C. Hardeman, J.R. Stehn, T. Böcking, P.W. Gunning, A small molecule inhibitor of tropomyosin dissociates actin binding from tropomyosin-directed regulation of actin dynamics, Scientific Reports 6, Art. No. 19816, 2016.

**Movie captions**

[Fig1A_H1975_FCS.mov; Fig1B_H1975_IGF.mov; Fig1C_H1975_Control.mov]

Entire time-lapse movies from which the snap shots in Figure 1 are derived.

[Fig3A_FCS_X; Fig3A_iEGFR_100nM_X; Fig3A_iEGFR_50nM_X]

Time-lapse movies from which the speed evolutions in Figure 3A were computed. The data comprised of three replicates for each condition (FCS, iEGFR 100nM and iEGFR 50nm).

Fig4A_StraightFronts.mov; Fig4B_Bridges.mov; Fig4C_UndulatingFronts.mov]

Movies showing simulated cell behavior for the parameter values given in Table 2. Snapshots of these movies are shown in Figure 4 A-C.
