# Supplementary material for: An individual-based model for collective cancer cell migration explains speed dynamics and phenotype variability in response to growth factors
Source: NPJ Syst Biol Appl. 2017 Mar 3;3:5. doi: 10.1038/s41540-017-0006-3 (PMC5460121; doi:10.1038/s41540-017-0006-3)
Supplement: Supplementary file 2 — Supplementary Movies [file 41540_2017_6_MOESM2_ESM.zip › NPJSBA-00155R1 Movie Files/movie file.docx]

**Movie captions**

Fig1A_H1975_FCS.mov

Fig1B_H1975_IGF.mov

Fig1C_H1975_Control.mov

Entire time-lapse movies from which the snap shots in Figure 1 are derived.

Fig3A_FCS_1, Fig3A_FCS_2, Fig3A_FCS_3

Fig3A_iEGFR_100nM_1, Fig3A_iEGFR_100nM_2, Fig3A_iEGFR_100nM_3

Fig3A_iEGFR_50nM_1, Fig3A_iEGFR_50nM_2, Fig3A_iEGFR_50nM_3

Time-lapse movies from which the speed evolutions in Figure 3A were computed. The data comprised of three replicates for each condition (FCS, iEGFR 100nM and iEGFR 50nm).

Fig4A_StraightFronts.mov

Fig4B_Bridges.mov

Fig4C_UndulatingFronts.mov

Movies showing simulated cell behavior for the parameter values given in Table 2. Snapshots of these movies are shown in Figure 4 A-C.
